# Determinants of Health and Physical Activity Levels Among Breast Cancer Survivors During the COVID-19 Pandemic: A Cross-Sectional Study

**DOI:** 10.3389/fphys.2021.624169

**Published:** 2021-02-05

**Authors:** Aline Rachel Bezerra Gurgel, Pedro Mingroni-Netto, Jose Carlos Farah, Christina May Moran de Brito, Anna S. Levin, Patricia Chakur Brum

**Affiliations:** ^1^School of Physical Education and Sport, University of São Paulo, São Paulo, Brazil; ^2^Centro de Práticas Esportivas da Universidade de São Paulo (CEPEUSP), São Paulo, Brazil; ^3^Instituto do Câncer do Estado de São Paulo, ICESP, Faculdade de Medicina da Universidade de São Paulo, São Paulo, Brazil; ^4^Department of Infectious Diseases and LIM49, Faculdade de Medicina, Universidade de São Paulo, São Paulo, Brazil

**Keywords:** physical activity, COVID-19, breast neoplasms, survivorship, pandemic (COVID-19)

## Abstract

**Background:**

Increased exercise and physical activity levels are recommended throughout cancer therapy and survivorship. Nonetheless, the COVID-19 pandemic and consequent social distancing are likely to cause a decline in physical activity.

**Objective:**

to evaluate the level of unsupervised physical activity of breast cancer survivors during the COVID-19 pandemic, and the factors associated with difficulties in engaging and maintaining recommended physical activity levels.

**Methods:**

This is a cross-sectional epidemiological study with a sample of 37 breast cancer survivors. They participated in a canoeing training program (project Remama) at the University of São Paulo before the COVID-19 pandemic. Socioeconomic aspects, engagement in physical activity, motivation, and potential exposure to COVID-19 were investigated through an online survey, administered in September of 2020.

**Results:**

During the pandemic, participants increased their body weight (5 ± 3.4 kg); 90% reported decreasing physical activity levels associated with increased sedentary time. Twenty-one (58%) participants exhibited some COVID-19-related symptoms, most used public transportation (59%), or returned to work during the period of a high incidence of COVID-19. The only factor associated with perceived difficulty in engaging in physical activities was having had more than three cancer treatments (RR: 2.14; 95% CI: 1.07–4.27).

**Conclusion:**

The COVID-19 pandemic led to a group of previously active breast cancer survivors to decrease their physical activity, gain weight, and have sedentary behavior. Specific tailored-care interventions are needed to prevent these occurrences, as overweight and physical inactivity may impose an additional risk for breast cancer recurrence and a severe course of COVID-19 in cancer patients.

## Introduction

A series of pneumonia cases of unknown etiology was reported in the city of Wuhan, China, in late 2019 (Xu et al., 2020). By sequencing a patient’s lower respiratory tract, a new virus was identified called severe acute respiratory syndrome coronavirus 2 (SARS-CoV-2) and its disease, coronavirus disease 2019 (COVID-19). It rapidly spread across countries and a new pandemic was declared by the World Health Organization (WHO) in March 2020. COVID-19 reached a mark of 20,162,474 cases and 737417 deaths globally in August of 2020 (World Health Organization, 2020). In Brazil, the Ministry of Health notified the first confirmed case on February 26, 2020. From that date until August 8, 2020, 3,012,412 cases were confirmed, and 100,477 deaths occurred as a result of COVID-19 in the country (Ministério da Saúde, 2020).

Social distancing recommendations significantly reduced levels of physical activity of the overall population and more profoundly in the population at increased risk, such as the elderly and those living with chronic non-communicable diseases (Damiot et al., 2020). Periods of confinement can be identified as a barrier to regular physical activity. A recent study showed that middle-aged individuals who adopt sedentary behavior have an increased risk of cancer mortality (Gilchrist et al., 2020). Physical inactivity may exacerbate comorbidities amongst older adults, including cardiovascular disease, cancer, and dysfunctional inflammatory responses (Flynn et al., 2019). In this scenario, older adults and individuals living with underlying conditions are at a greater risk for complications during COVID-19 disease (Damiot et al., 2020).

Worldwide, the incidence of cancer and mortality remain high. Over 18 million new cases were registered in 2018, alongside 9.6 million deaths (Bray et al., 2018). Among women, the most diagnosed neoplasm is breast cancer (excluding non-melanoma skin cancer), which also represents the major oncological cause of death in this population, with 2.1 million diagnosed cases in 2018, accounting for almost one in four cancer cases (Bray et al., 2018). Despite the escalating cancer incidence, advances in early diagnosis and breast cancer therapy improved five-year overall survival rates, now exceeding 90% when diagnosed in the early stages (Simon et al., 2019; Siegel et al., 2020).

In this context, particular attention should be dedicated to metabolic aspects including weight control and management of physical inactivity, through lifestyle interventions. In fact, increased physical activity levels, exercise, and body weight control have been considered strong allies to cancer patients and survivors, as they positively impact physical capacities, fatigue, depressive symptoms, anxiety, and quality of life (Campbell et al., 2019; Mctiernan et al., 2019; Nardin et al., 2020). Since the literature of the past two decades has provided evidence that supports the practice of physical activity during and after cancer treatment (Schmitz et al., 2019); and that physical activity helps to prevent neoplastic recurrence (Patel et al., 2019), it is of utmost importance to develop public health strategies to encourage this practice.

The present study aimed to evaluate the level of unsupervised physical activity of breast cancer survivors during the COVID-19 pandemic, and the factors associated with difficulties in engaging and maintaining recommended physical activity levels.

## Materials and Methods

This is a cross-sectional study designed to address physical activity levels in a convenience sample of breast cancer survivors. All subjects of this study participate in a group canoeing training project called Remama at the University of São Paulo, in which they are longitudinally followed-up.

Remama is a collaborative project between the Cancer Institute of the State of Sao Paulo, the School of Physical Education of the University of São Paulo (USP), and the Sports Center of USP. The inclusion criteria were participants who have finished their prescribed breast cancer treatment with curative intent, including surgery, systemic cytotoxic chemotherapy, and radiotherapy; who are aged between 35 and 75 years old; and have concluded their treatment within a time span of at least 6 months up to 3 years. The exclusion criteria were patients who had metastatic disease, severe lymphedema, organic dysfunction, or uncontrolled risk factors (hypercholesterolemia, diabetes, hypertension).

Previously to the pandemic, Remama participants received a physical activity recommendation booklet (based on WHO guidelines). The booklet suggests different exercise modalities such as aerobic, strength and flexibility, and an increase in overall physical activity level as part of a behavioral change. It also recommends precautions participants should adopt while exercising. Due to the COVID-19 pandemic, access to public and private spaces is restricted. The participants were instructed to increase physical activity levels at home because of the discontinuation of face-to-face training sessions. We sent online questionnaires to 41 Remama participants in September 2020. The participants were instructed through videoconference.

### Instruments

The level of physical activity, sport, and leisure was assessed using an instrument built especially for this study and adapted from the Minnesota Leisure Time Physical Activity Questionnaire (Taylor et al., 1978), that is a widely used tool to address physical activity levels in different populations (Elosua et al., 2000; Lozano-Lozano et al., 2016) and that has been validated for Brazilian population (Lustosa et al., 2011). It quantified: (a) the time that participants spent on accumulated physical activities in daily life (lifestyle), called “not programmed movement”; (b) the time spent on physical activities to meet the goal of 150 min per week of physical exercise; and (c) the total time they spent sitting, whether in leisure activities (such as watching television, cell phones, etc.) or in professional work-related activities.

To assess current patterns of physical activity upon the COVID-19 pandemic, a survey adapted from a questionnaire used by Lesser and Nienhuis (2020) was applied. This was based on the Nature Relatedness scale (Nguyen and Brymer, 2018) and on the Godin Leisure Questionnaire, which has been validated for the Brazilian population (São-João et al., 2013). This included questions on the (a) the importance of carrying out outdoor activities (Nisbet et al., 2009); (b) sedentary behavior, questions based on a study by Ekelund et al. (2016); and, (c) the Behavioral Regulations in Exercise questionnaire (BREQ-3), also validated to adult Brazilian population (Guedes and Sofiati, 2015) to assess the participants’ motivation for physical activity at home (Rutten et al., 2014). Participants were asked to answer questions related to motivation and training opportunities, indicating answers on eight statements using a 5-point Likert scale ranging from 1 (strongly disagree) to 5 (strongly agree). Finally, we also investigated the potential exposure to SARS-CoV-2. A validated questionnaire adapted from the Mount Sinai Hospital (2020) survey was administered. The participants were asked to answer the questions considering the period starting on March 1, 2020.

#### Dependent Variable

Participants were asked about adherence to physical activity in the context of measuring an individuals’ motivation, support, and opportunity to engage in physical activity. Each answer was scored on the Likert scale from one (strongly disagree), two (partially disagree), three (neutral), four (partially agree) to five (strongly agree). Next, we generated dichotomous variables from the Likert scale and used them for measuring the participants’ difficulty in engage with physical activity. The scores 1 and 2 were accounted as “no,” while the scores from three to five were considered “yes.”

#### Independent Variables

Demographic data were evaluated, including age, education, occupation, return to work, and professional working routine. Clinical data were retrieved on body mass index, menopausal status, presence of underlying conditions, and cancer treatment adopted (chemotherapy, radiotherapy, surgery, immunotherapy, and hormone-based therapy). Behavioral factors included opportunity and motivation for physical activity. Lastly, patients were asked whether they had experienced any symptoms of COVID-19 during social distancing.

### Statistical Analyses

Two groups were compared: participants who did experience difficulty in engaging in physical activity against those who did not. Results are presented as relative risk (RR) and 95% confidence intervals (CI). A 95% CI that did not include 1.00 was considered statistically significant. Statistical analysis was performed using EpiInfo^TM^ version 7.2.4.0.

### Ethical Considerations

This study was approved by the School of Physical Education Research Ethical Board (CAAE: 12242919.9.0000.5391) in accordance with the 1964 Declaration of Helsinki amendment in 2013. All participants signed a consent form. The identity and individual information of the subjects are confidential, and the data were analyzed in an aggregate form. The raw data supporting the conclusions of this article will be made available by the authors upon request.

## Results

The survey was sent to 41 members of the project Remama of which 37 responded (90%). The demographic and clinical data and, the characteristics of their breast cancer can be seen in Table 1.

**TABLE 1:** Demographic and clinical characteristics, and responses of 37 breast cancer survivors, enrolled in a program for physical activity, who answered a survey on their level of activity and perceptions on the subject, on possible COVID-19 symptoms, and on potential exposure to the disease during the pandemic.

Age, years, mean ± SD	57 ± 7.4
**Ethnicity *n* (%)**	
White	19(51%)
Black/brown	13(35%)
Others	5(14%)
**Education *n* (%)**	
Up to high school	15(41%)
University	12(32%)
Post-graduation	10(27%)
Menopausal *n* (%)	22(59%)
**Obesity status *n* (%)**	
Overweight	22(59%)
Weight unchanged	7(19%)
Weight loss	6(16%)
Unknown	2(5%)
Weight gain (Kg), median (range)	3.75 (1–15)
**Type of treatment *n* (%)**	
Surgery	32(86%)
Chemotherapy	32(86%)
Radiotherapy	31(84%)
Endocrine	12(32%)
Immunotherapy	2(5%)
**Type of malignancy *n* (%)**	
Invasive ductal carcinoma	15(41%)
*In situ* ductal carcinoma	5(14%)
Invasive lobular carcinoma	3(8%)
Unknown	10(27%)
Other	4(11%)
Time since treatment completion, months, median (range)	46 (1–95)
Use of tamoxifen *n* (%)	15(41%)
Under cancer treatment *n* (%)	4(11%)
Current treatments *n* (%) Systemic arterial hypertension	11(30%)
Diabetes mellitus	5(14%)
**Symptoms suggestive of COVID-19 *n* (%)**	
No symptoms	16(43%)
Headache	13(35%)
Myalgia	7(19%)
Cough	5(14%)
Coryza	5(14%)
Sore throat	4(11%)
Other	5(14%)
Duration of symptoms (days), median (range)	4 (4–20)
Admitted to hospital *n* (%)	3(8%)
Contact with suspected or confirmed case of COVID-19 at home *n* (%)	5(14%)
Duration of exposure (days), median (range)	3 (3–10)
Contact with suspected or confirmed case of COVID-19 outside the home *n* (%)	8(22%)
Worked outside the home *n* (%)	13(35%)
Number of times per week worked outside the home, median (range)	3.5 (1–6)
Used public transportation *n* (%)	22(59%)
Number of times per week used public transportation, median (range)	2 (0–6)
**Levels and characteristics of physical activity during the COVID-19 pandemic**	
Prefers outdoor training *n* (%)	22(59%)
Considers outdoor activities very important *n* (%)	25(68%)
Reduced physical activity levels during the pandemic *n* (%)	33(89%)

*SD, standard deviation.*

The mean age of the volunteers was 57 years old, with the majority aged 55 years or older. Twenty-two (59%) participants reported increase in bodyweight (5 kg, ranging from 1 to 15 kgs). The five most frequently reported symptoms were headache, myalgia, cough, coryza, and sore throat. These symptoms occurred mainly in July and lasted a few days. Although three participants were hospitalized, no subject from the population developed severe COVID-19 or post-COVID19 complications.

**FIGURE 1:**
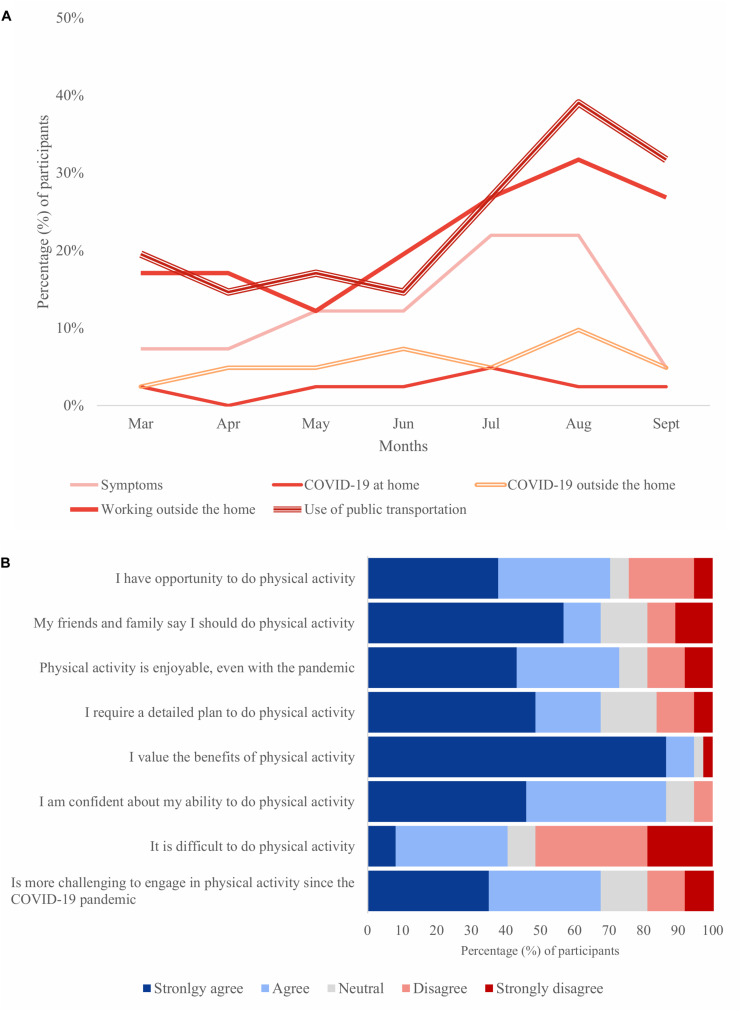
Participants’ behavior outside and inside the home during the COVID-19 pandemic. **(A)** Proportion of participants who reported exposure, from March to September 2020, to suspected or confirmed cases of COVID-19 at home, outside the home, and who used public transportation (*n* = 37). **(B)** Barriers and facilitators for physical activity perceived in September 2020 (*n* = 37). Results are presented in percentage.

Participants worked outside the home mainly in July, August, and September, roughly between three and four times a week. This coincides with the use of public transportation in July, August, and September. They used public transportation between two and three times a week. Figure 1A summarizes participants’ potential exposure to SARS-CoV-2 reported from March to September 2020.

Barriers and facilitators of physical activity perceived by the participants can be seen in Figure 1B. The vast majority reported that it is challenging to engage in physical activity since the pandemic, although most had the opportunity and valued the effect of exercise and being physically active.

The levels of programmed and not programmed movement are described in Table 2. Most volunteers reported having adopted alternative movement and used stairs. Besides, the majority stated they performed housework activities. Roughly half of them achieved 150 min/week of physical activity in September 2020. As to sedentary behavior, 20 (54%) participants reported remaining seated for over two consecutive hours during the week, and 19 (51%), during the weekend.

**TABLE 2:** Evaluation of programmed and non-programmed physical activities of breast cancer survivors enrolled in a program for physical activity, who answered a survey on their level of activity, (*n* = 37).

**Non-programmed movement/daily activities**	
Adopted alternative movement *n* (%)	28(76%)
Used stairs *n* (%)	26(70%)
Walked *n* (%)	24(65%)
Worked in the last 2 weeks *n* (%)	25(68%)
Movement in professional activities *n* (%)	16(43%)
Movement in leisure activities *n* (%)	15(41%)
Performed housework *n* (%)	31(84%)
Felt discomfort during housework activities *n* (%)	11(30%)
**Programmed activities**	
Achieved 150 min/week physical activity *n* (%)	18(49%)
**Sedentary behavior during week/weekend**	
Remained seated for more than two consecutive hours during week *n* (%)	20(54%)
Remained seated for more than two consecutive hours during weekend *n* (%)	19(51%)

The potential association between the patients having reported having difficulty engaging in physical activities can be seen in Table 3. Women who underwent at least three anticancer treatments found difficulty in doing physical activity.

**TABLE 3:** Evaluation of factors potentially associated with the perception of the patients that it is difficult to engage in physical activities (Survey taken during the COVID-19 pandemic with breast cancer survivors previously enrolled in a canoeing program, *n* = 37).

Find it difficult to engage in physical activity			

	Yes (%) *n* = 15	No (%) *N* = 22	RR (CI 95%)
Age > 55 years old	9 (60%)	12 (55%)	1.14 (0.51–2.55)
Obesity	11 (73%)	14 (64%)	1.03 (0.53–3.29)
Studied up to high school	9 (40%)	13 (59%)	1.02 (0.46–2.27)
Menopause	6 (40%)	16 (73%)	0.75 (0.32–1.78)
Has hypertension	5 (33%)	6 (27%)	1.18 (0.52–2.65)
Had > 3 cancer treatments	5 (33%)	2 (9%)	2.14 (1.07–4.27) *
Prefers outdoor activities	8 (53%)	14 (64%)	0.72 (0.33–1.55)
Has opportunity to do physical activity	9 (60%)	18 (82%)	0.55 (0.26–1.15)
Works outside the home	7 (47%)	6 (27%)	1.60 (0.75–3.44)
Presented COVID-19 symptoms	9 (60%)	12 (55%)	1.14 (0.51–2.55)

*Statistically significant.

## Discussion

Our survey with 37 breast cancer survivors revealed that most of the participants reduced their physical activity level and gained weight upon temporary suspension of the canoeing training project Remama at COVID-19 pandemic onset. Although no factors were associated with reducing activity, our study discloses that having been submitted to more than three cancer treatments was associated with their perception of difficulty to do physical activity. Taken together, these aspects may impose an additional risk of a severe course of COVID-19 with a worse prognosis. No other modifiable factor could be associated with this outcome. This finding was crucial to support our research group in developing a new strategy to engage participants in a physical activity program that is active *via* online classes currently.

Exercise, preferably following an exercise program (Newton et al., 2020), can improve outcomes in people who have or have had cancer, such as well-being, body weight control, and reduce the cancer recurrence risk. Bodyweight gain and physical inactivity are known to increase the cancer recurrence risk (Meyerhardt et al., 2006; Renehan et al., 2008), cardiovascular disease, and metabolic disorders (Ford and Caspersen, 2012). Physical activity enhances quality of life and improves the effectiveness of therapies, mitigating potential adverse effects inherent to antineoplastic therapy and drug toxicity. Furthermore, it may minimize or reverse the progression of other chronic diseases. Besides that, cancer patients may be at an increased risk of developing severe COVID-19 due to the presence of underlying conditions, anticancer therapy, and old age, among others (Galluzzi et al., 2015; Dai et al., 2020; Damiot et al., 2020).

In our study, most women reported they preferred outdoor activities, and the majority considered outdoor spaces especially important for physical activity. However, due to the pandemic, the health authorities restricted these outdoor activities. This may have been one of the contributing factors to our results. A larger number of cancer treatments was significantly associated with perceived difficulty in being physically active. This might be due to the long-term side effects associated with antineoplastic therapies. This includes fatigue, insomnia, persistent pain, lymphedema, among other unwanted effects that might impose barriers to physical activity engagement (Campbell et al., 2019). In fact, the probability of developing long-term side effects increases as the number of antineoplastic treatment increases (Cheville, 2001; Stout and Sabo Wagner, 2019).

A large proportion of the participants reported having had symptoms, mostly during July and August, the months with the highest incidence of COVID-19 in São Paulo. This was also the period in which the participants reported more frequent use of public transportation and return to work. Many of our participants were self-employed. They may have found themselves pushed back to work to provide for their families. This behavior possibly increased the chance of exposure to symptomatic or asymptomatic people outside the home. Brazilian women who are workers in the informal economy were disproportionally affected by the pandemic (International Labour Organization, 2020a), as they do not have access to social protection mechanisms. Thus, we believe that our study reflects a particular socioeconomic scenario that exacerbates social inequality. This scenario differs from that of other countries that provide social protection, allowing the population to stay at home and follow social distancing orders, minimizing the spread of SARS-CoV-2. We believe that adequate responses to the pandemic should observe the particularities of each country, and policies ensuring measures for social health protection and extending financial protection should prioritize vulnerable workers (International Labour Organization, 2020b).

Our study has limitations. Self-reported physical activity levels may impose imprecision on the data. Furthermore, this survey does not fully report all aspects of BREQ-3 or Minnesota questionnaire, including psychological aspects. The interpretation of our findings might be limited by the sample size. Nevertheless, understanding the practice of physical activity in the context of the life cycle and macro determinants of behavior is of vital importance (Hallal, 2014). Although using a small sample, this study provides fresh insights into a complex problem and may provide a direction for interventions. In this sense, alternative ways of delivering supervised and structured physical activity (exercise) based on telehealth should be studied as an alternative for increasing the adherence to exercise aiding to maintain an active lifestyle in the pandemic and even afterward (Newton et al., 2020).

In conclusion, previously active breast cancer survivors found themselves inactive or with reduced physical activity levels and gained weight during the pandemic. Those who underwent multiple antineoplastic treatments found it difficult to engage in physical activity. Therefore, our study calls for tailored-care interventions and alternative ways of delivering supervised exercise to cancer survivors during the COVID-19 pandemic and beyond.

## Data Availability Statement

The raw data supporting the conclusions of this article will be made available by the authors, without undue reservation.

## Ethics Statement

The studies involving human participants were reviewed and approved by Research Ethics Committee of School of Physical Education and Sports of University of São Paulo. The patients/participants provided their written informed consent to participate in this study.

## Author Contributions

PB, AL, and AG planned the design of the study and data collection. PM-N carried out data collection through an online survey. AG and PM-N worked on data organization and treatment. AL provided guidance on statistical analysis. PB and AL contributed to the interpretation of the results. AG drafted the manuscript and designed the figures. JF is the coordinator of the Cancer institute rehabilitation program supporting Remama group health care. AG and PB drafted the manuscript with critical revision from AL and CB. All authors provided critical feedback on the present manuscript.

## Conflict of Interest

The authors declare that the research was conducted in the absence of any commercial or financial relationships that could be construed as a potential conflict of interest.
