# Corrigendum: Determinants of Health and Physical Activity Levels Among Breast Cancer Survivors During the COVID-19 Pandemic: A Cross-Sectional Study

**DOI:** 10.3389/fphys.2021.719688

**Published:** 2021-07-26

**Authors:** Aline Rachel Bezerra Gurgel, Pedro Mingroni-Netto, Jose Carlos Farah, Christina May Moran de Brito, Remama Group, Anna S. Levin, Patricia Chakur Brum

**Affiliations:** ^1^School of Physical Education and Sport, University of São Paulo, São Paulo, Brazil; ^2^Centro de Práticas Esportivas da Universidade de São Paulo (CEPEUSP), São Paulo, Brazil; ^3^Instituto Do Câncer Do Estado de São Paulo, ICESP, Faculdade de Medicina da Universidade de São Paulo, São Paulo, Brazil; ^4^Department of Infectious Diseases and LIM49, Faculdade de Medicina, Universidade de São Paulo, São Paulo, Brazil

**Keywords:** physical activity, COVID-19, breast neoplasms, survivorship, pandemic (COVID-19)

In the original article, the reference for “**A series of pneumonia cases of unknown etiology was reported in the city of Wuhan, China, in late 2019**” was incorrectly written as **Taylor, H. L., Jacobs, D. R., Schucker, B., Knudsen, J., Leon, A. S., and Debacker, G. (1978). A questionnaire for the assessment of leisure time physical activities**. ***J Chronic Dis***. **31, 741–755**. **doi: 10.1016/0021-9681(78)90058-9**. It should be **Xu, X., Chen, P., Wang, J., Feng, J., Zhou, H., Li, X., et al. (2020). Evolution of the novel coronavirus from the ongoing Wuhan outbreak and modeling of its spike protein for risk of human transmission**. ***Sci. China Life Sci***. **63, 457–460**. **doi: 10.1007/s11427-020-1637-5**.

In the original article, the reference for “**COVID- 19 reached a mark of 20,162,474 cases and 737417 deaths globally in August of 2020**” was incorrectly written as **Elosua, R., Garcia, M., Aguilar, A., Molina, L., Covas, M.-I., Marrugat, J., et al. (2000). Validation of the minnesota leisure time Spanish women**. ***Med. Sci. Sports Exerc***. **32, 1431–1437**. **doi: 10.1097/00005768-200008000-00011**. It should be **World Health Organization (2020)**. ***Coronavirus Disease 2019***. **Situation Report – 205. Available online at:** **https://www.who.int/docs/default-source/coronaviruse/situation-reports/20200812-covid-19-sitrep-205.pdf?sfvrsn=627c9aa8_2** **(accessed August 23, 2020)**.

In the original article, the reference for “**From that date until August 8, 2020, 3,012,412 cases were confirmed, and 100,477 deaths occurred as a result of COVID-19 in the country**” was incorrectly written as **Lozano-Lozano, M., Mart**í**n-Mart**í**n, L., Galiano-Castillo, N., Álvarez-Salvago, F., Cantarero-Villanueva, I., Fernández-Lao, C., et al. (2016). Integral strategy to supportive care in breast cancer survivors through occupational therapy and a m-health system: design of a randomized clinical trial**. ***BMC Med. Inform. Decis***. ***Mak***. **16:150**. **doi: 10.1186/s12911-016-0394-0**. It should be **Ministério da Saúde (2020)**. ***Boletim*** ***Epidemiológico Especial***. **Doença pelo Coronavírus COVID-19. Available online at:** **http://antigo.saude.gov.br/images/pdf/2020/August/12/Boletim-epidemiologico-COVID-26.pdf** **(accessed August 23, 2020)**.

In the original article, the reference for “**Social distancing recommendations significantly reduced levels of physical activity of the overall population and more profoundly in the population at increased risk, such as the elderly and those living with chronic non-communicable diseases**” was incorrectly written as **Lustosa, L. P., Pereira, D. S., Dias, R. C., Britto, R. R., Parentoni, A. N., Pereira, L. S. M., et al. (2011). Tradução e adaptação transcultural do minnesota leisure time activities questionnaire em idosos**. ***Geriatr. Gerontol. Aging*** **5, 57–65**. It should be **Damiot, A., Pinto, A. J., Turner, J. E., and Gualano, B. (2020). Immunological implications of physical inactivity among older adults during the COVID-19 pandemic**. ***Gerontology*** **(2020) 66, 431–438**. **doi: 10.1159/000509216**.

In the original article, the reference for “**A recent study showed that middle-aged individuals who adopt sedentary behavior have an increased risk of cancer mortality**” was incorrectly written as **Lesser, I. A., and Nienhuis, C. P. (2020). The impact of COVID-19 on physical activity behavior and well-being of canadians**. ***Int. J. Environ. Res. Public Health*** **17:3899**. **doi: 10.3390/ijerph17113899**. It should be **Gilchrist, S. C., Howard, V. J., Akinyemiju, T., Judd, S. E., Cushman, M., Hooker, S. P., et al. (2020). Association of sedentary behavior with cancer mortality in middle-aged and older US adults**. ***JAMA Oncol***. **6, 1210–1217**. **doi: 10.1001/jamaoncol.2020.2045**.

In the original article, the reference for “**Physical inactivity may exacerbate comorbidities amongst older adults, including cardiovascular disease, cancer, and dysfunctional inflammatory responses**” was incorrectly written as **Nguyen, J., and Brymer, E. (2018). Nature-based guided imagery as an intervention for state anxiety**. ***Front. Psychol***. **9:1858**. **doi: 10.3389/fpsyg.2018.01858**. It should be **Flynn, M. G., Markofski, M. M., and Carrillo, A. E. (2019). Elevated inflammatory status and increased risk of chronic disease in chronological aging: inflamm-aging or inflamm-inactivity?** ***Aging Dis***. **(2019) 10, 147–156**. **doi: 10.14336/AD.2018.0326**.

In the original article, the reference for “**In this scenario, older adults and individuals living with underlying conditions are at a greater risk for complications during COVID-19 disease**” was incorrectly written as **Lustosa, L. P., Pereira, D. S., Dias, R. C., Britto, R. R., Parentoni, A. N., Pereira, L. S. M., et al. (2011). Tradução e adaptação transcultural do minnesota leisure time activities questionnaire em idosos**. ***Geriatr. Gerontol. Aging*** **5, 57–65**. It should be **Damiot, A., Pinto, A. J., Turner, J. E., and Gualano, B. (2020). Immunological implications of physical inactivity among older adults during the COVID-19 pandemic**. ***Gerontology*** **66, 431–438**. **doi: 10.1159/000509216**.

In the original article, the reference for “**Worldwide, the incidence of cancer and mortality remain high. Over 18 million new cases were registered in 2018, alongside 9.6 million deaths**” was incorrectly written as **São-João, T. M., Rodrigues, R. C. M., Gallani, M. C. B. J., Miura, C. T. D. P., Domingues, G. D. B. L., and Godin, G. (2013). Cultural adaptation of the Brazilian version of the godin-shephard leisure-time physical activity questionnaire**. ***Rev. Saude*** ***Publica*** **47, 3**. **doi: 10.1590/S0034-8910.2013047003947**. It should be **Bray, F., Ferlay, J., Soerjomataram, I., Siegel, R. L, Torre, L. A., and Jemal, A. (2018). Global cancer statistics 2018: GLOBOCAN estimates of incidence and mortality worldwide for 36 cancers in 185 countries**. ***CA Cancer J. Clin***. **(2018) 68, 394–424**. **doi: 10.3322/caac.21492**.

In the original article, the reference for “**Among women, the most diagnosed neoplasm is breast cancer (excluding non-melanoma skin cancer), which also represents the major oncological cause of death in this population, with 2.1 million diagnosed cases in 2018, accounting for almost one in four cancer cases**” was incorrectly written as **São-João, T. M., Rodrigues, R. C. M., Gallani, M. C. B. J., Miura, C. T. D. P., Domingues, G. D. B. L., and Godin, G. (2013). Cultural adaptation of the Brazilian version of the godin-shephard leisure-time physical activity questionnaire**. ***Rev. Saude*** ***Publica*** **47, 3**. **doi: 10.1590/S0034-8910.2013047003947**. It should be **Bray, F., Ferlay, J., Soerjomataram, I., Siegel, R. L, Torre, L. A., and Jemal, A. (2018). Global cancer statistics 2018: GLOBOCAN estimates of incidence and mortality worldwide for 36 cancers in 185 countries**. ***CA Cancer J. Clin***. **(2018) 68, 394–424**. **doi: 10.3322/caac.21492**.

In the original article, the reference for “**Despite the escalating cancer incidence, advances in early diagnosis and breast cancer therapy improved five-year overall survival rates, now exceeding 90% when diagnosed in the early stages**” was incorrectly written as **Nisbet, E. K., Zelenski, J. M., and Murphy, S. A. (2009). The nature relatedness scale: linking individuals' connection with nature to environmental concern and behavior**. ***Environ. Behav***. **41, 715–740**. **doi: 10.1177/0013916508318748;** **Ekelund, U., Steene-Johannessen, J., Brown, W. J., Fagerland, M. W., Owen, N., Powell, K. E., et al. (2016). Does physical activity attenuate, or even eliminate, the detrimental association of sitting time with mortality? A harmonised meta-analysis of data from more than 1 million men and women**. ***Lancet*** **388:10051**. **doi: 10.1016/S0140-6736(16)30370-1**. It should be **Simon, S. D., Bines, J., Werutsky, G., Nunes, J. S., Pacheco, F. C., Segalla, J. G., et al. (2019). Characteristics and 238 prognosis of stage I-III breast cancer subtypes in Brazil: the AMAZONA retrospective cohort 239 study**. ***Breast*** **44, 113–119**. **doi: 10.1016/j.breast.2019.01.008;** **Siegel, R. L., Miller, K. D., and Jemal, A. (2020). Cancer statistics, 2020**. ***CA Cancer J. Clin***. **70, 7–30**. **doi: 10.3322/caac.21590**.

In the original article, the reference for “**In fact, increased physical activity levels, exercise, and body weight control have been considered strong allies to cancer patients and survivors, as they positively impact physical capacities, fatigue, depressive symptoms, anxiety, and quality of life**” was incorrectly written as **Guedes, D. P., and Sofiati, S. L. (2015). Translation and psicometric validation of the behavioral regulation in exercise questionnaire for use in Brazilian adults**. ***Rev. Bras. Ativ. Fís. Saúde*** **20, 397–412**. **doi: 10.12820/rbafs.v.20n4p397;** **Rutten, G. M., Meis, J. J. M., Hendriks, M. R. C., Hamers, F. J. M., Veenhof, C., and Kremers, S. P. J. (2014). The contribution of lifestyle coaching of overweight patients in primary care to more autonomous motivation for physical activity and healthy dietary behaviour: results of a longitudinal study**. ***Int. J. Behav. Nutr. Phys. Act***. **11:86**. **doi: 10.1186/s12966-014-0086-z;** and **Mount Sinai Hospital (2020)**. ***COVID-19 Symptoms***. **Available online at:** **https://www.mountsinai.org/health-library/symptoms/covid-19-symptoms** **(Accessed August, 2015)**. It should be **Campbell, K. L., Winters-Stone, K. M., Wiskemann, J., May, A. M., Schwartz, A. L., Courneya, K. S., et al. (2019). Exercise guidelines for cancer survivors: consensus statement from international multidisciplinary roundtable**. ***Med. Sci. Sports Exerc***. **51, 2375–2390. **doi: 10.1249/MSS.0000000000002116;** Nardin, S., Mora, E., Varughese, F. M., D'Avanzo, F., Vachanaram, A. R., Rossi, V., et al. (2020). Breast cancer survivorship, quality of life, and late toxicities**. ***Front. Oncol***. **(2020) 10:864**. **doi: 10.3389/fonc.2020.00864;** and **Mctiernan, A., Friedenreich, C. M., Katzmarzyk, P. T., Powell, K. E., Macko, R., Buchner, D., et al. (2019). Physical activity in cancer prevention and survival: a systematic review**. ***Med. Sci. Sports Exerc***. **(2019) 51, 1252–1261**. **doi: 10.1249/MSS.0000000000001937**.

In the original article, the reference for “**Since the literature of the past two decades has provided evidence that supports the practice of physical activity during and after cancer treatment**” was incorrectly written as **Newton, R. U., Hart, N. H., and Clay, T. (2020). Keeping patients with cancer exercising in the age of COVID-19**. ***JCO*** ***Oncol. Pract***. **16, 656–664**. **doi: 10.1200/OP.20.00210**. It should be **Schmitz, K. H., Campbell, A. M., Stuiver, M. M., Pinto, B. M., Schwartz, A. L., Morris, G. S., et al. (2019). Exercise is medicine in oncology: engaging clinicians to help patients move through cancer**. ***CA Cancer J. Clin***. **(2019) 69, 468–484**. **doi: 10.3322/caac.21579**.

In the original article, the reference for “**and that physical activity helps to prevent neoplastic recurrence**” was incorrectly written as **Renehan, A. G., Roberts, D. L., and Dive, C. (2008). Obesity and cancer: pathophysiological and biological mechanisms**. ***Arch. Physiol. Biochem***. **114, 71–83**. **doi: 10.1080/13813450801954303**. It should be **Patel, A. V., Friedenreich, C. M., Moore, S. C., Hayes, S. C., Silver, J. K., Campbell, K. L., et al. American College of Sports Medicine roundtable report on physical activity, sedentary behavior, and cancer prevention and control**. ***Med. Sci. Sports Exerc***. **(2019) 51, 2391–2402**. **doi: 10.1249/MSS.0000000000002117**.

In the original article, the reference for “**This behavior possibly increased the chance of exposure to symptomatic or asymptomatic people outside the home. Brazilian women who are workers in the informal economy were disproportionally affected by the pandemic**” was incorrectly written as **International Labour Organization (2018)**. ***COVID-19 and the World of Work: Impact and Policy Responses***. **Available online at:** **https://www.ilo.org/wcmsp5/groups/public/---dgreports/---dcomm/documents/briefingnote/wcms_738753.pdf** **(accessed September, 2020)**. It should be **International Labour Organization (2020)**. ***COVID-19 and the World of Work: Impact and Policy Responses***. **Available online at:** **https://www.ilo.org/wcmsp5/groups/public/---dgreports/---dcomm/documents/briefingnote/wcms_738753.pdf** **(accessed September, 2020)**.

The authors apologize for this error and state that this does not change the scientific conclusions of the article in any way. The original article has been updated.

## Publisher's Note

All claims expressed in this article are solely those of the authors and do not necessarily represent those of their affiliated organizations, or those of the publisher, the editors and the reviewers. Any product that may be evaluated in this article, or claim that may be made by its manufacturer, is not guaranteed or endorsed by the publisher.

