# Supplementary material for: Determinants of Health and Physical Activity Levels Among Breast Cancer Survivors During the COVID-19 Pandemic: A Cross-Sectional Study
Source: Front Physiol. 2021 Feb 5;12:624169. doi: 10.3389/fphys.2021.624169 (PMC7893086; doi:10.3389/fphys.2021.624169)
Supplement: Supplementary file 1 [file Table_1.DOCX]

Supplementary Material

Proportion of participants who reported occurrence of symptoms suggestive of COVID-19, exposure to suspected or confirmed cases of COVID-19 at home, outside the home, and who used public transportation, from March to September 2020 (n=37).

|  | **March** | **April** | **May** | **June** | **July** | **August** | **September** |
| --- | --- | --- | --- | --- | --- | --- | --- |
| Time of occurrence of COVID-19 symptoms n (%) | 3 (8%) | 3 (8%) | 5 (14%) | 5 (14%) | 9 (24%) | 9 (24%) | 2 (5%) |
| Time of exposure to a suspected or confirmed case of COVID-19 outside the home n (%) | 1 (3%) | 2 (5%) | 2 (5%) | 3 (8%) | 2 (5%) | 4 (11%) | 2 (5%) |
| Months when worked outside the home n (%) | 7 (19%) | 7 (19%) | 5 (14%) | 8 (22%) | 11 (30%) | 13 (35%) | 11 (30%) |
| Months when used public transportation n (%) | 8 (22%) | 6 (16%) | 7 (19%) | 6 (16%) | 11 (30%) | 16 (43%) | 13 (35%) |
